# Relationship between High Expression of Kaiso Protein and Poor Prognosis of Lung Cancer and the Regulation Mechanism of Malignant Phenotype of Lung Cancer Cells

**DOI:** 10.1155/2021/7388368

**Published:** 2021-12-22

**Authors:** Shasha Zhu, Ning Zhou, Ning Ding, Shanshan Li, Xiaoxing Liu, Guangming Ren, Qingling Li, Min Zhou

**Affiliations:** The Department of Respiratory, The Affiliated Xuzhou First People's Hospital of Xuzhou Medical University, Xuzhou, Jiangsu, China

## Abstract

In this study, Kaiso was discovered to be a unique member of the POZ-zinc fingers family of transcription factors, which has been implicated in the genesis and progression of cancer. Although there is still some debate, Kaiso is believed to be implicated in the development of human cancer. It should be noted that there is minimal evidence available on the therapeutic relevance of nuclear Kaiso in lung cancer in humans. Histone or DNA modifications that control gene activity outside of the underlying sequence are examples of epigenetic alternations. Epigenetic alterations are heritable but reversible. Human illness, such as lung cancer, is often related to epigenetic dysregulation. In preclinical and clinical studies, epigenetic-targeted therapy has shown significant therapeutic promise for solid tumours and has been used in the treatment of haematological malignancies using different medicines targeting epigenetic regulators. It is important to note that the abnormal activities of Kaiso enzymes in tumour growth are summarised below and the development of inhibitors or medicines targeting epigenetic enzyme regulation is highlighted.

## 1. Introduction

Lung cancer is both the most common diagnosed cancer and the leading cause of cancer-related deaths in China. During the past three decades, the incidence and mortality of lung cancer in China have been increasing rapidly. According to data from National Central Cancer Registry (NCCR) in 2010, the crude incidence of lung cancer in China was 46.08 per 100,000 population (61.86 per 100,000 men and 29.54 per 100,000 women), with an estimated over 600,000 new diagnosed lung cancer patients (416,333 males and 189,613 females). Meanwhile, the crude mortality of lung cancer in China was 37.00 per 100,000 population (50.04 per 100,000 men and 23.33 per 100,000 women). Consistent with the change in developed countries, adenocarcinoma has become the most predominant histological subtype of lung cancer in China. Kaiso belongs to the BTB/POZ (Broad-Complex, Tramtrack, and Bric-a-brac/Poxvirus and Zinc finger) family of transcriptional repressors. At one end is the carboxyl-terminal DNA-binding C2H2 zinc finger domain, while at the other is the BTB/POZ protein-protein interaction domain. Only the POZ-ZF transcription factor Kaiso has been shown to bind DNA in both orientations so far. Matrilysin, cyclin D1, and c-myc have all been identified as potential Kaiso targets, and the zinc finger domain seems to be involved in their regulation. Kaiso may have a role in tumour development, but further research is needed to confirm this. One study found that suppressing cancer-associated Wnt target genes including matrilysin and Wnt11 made Kaiso an effective tumour suppressant. The reverse is true when compared to the results obtained from Kaiso-null mice. Researchers created mice resistant to intestinal carcinogenesis by breeding Apc^Min/+^ tumour-susceptible females with Kaiso-deficiency males. In other words, Kaiso may be an “opportunistic” oncogene reliant on methylation, inhibiting the tumour-suppressive CDKN2A gene and giving colon cancer cells an edge over healthy cells. Regardless of the ongoing debate, there is a direct connection between Kaiso and cancer in people. There have been a few clinicopathological studies that have looked at the connection between Kaiso expression and human malignancy, such as lung cancer, but further study is required. To begin, Soubry A. et al. examined the expression pattern of Kaiso in human tissues using immunohistochemistry. Human malignant and noncancer tissues both contained this transcription factor in their cytoplasm, as opposed to cultured cells, where it was found mostly in the nucleus (such as MDCK, NIH3T3, HT29, and SW48). Since Kaiso's subcellular localization seems to be changing over time rather than being static, it is possible the microenvironment is playing a role. There needs to be further study to determine whether Kaiso is expressed in lung cancer and if its subcellular location is associated with the grade or prognosis of the tumour. We need to learn more about the transcription factor's function in the cytoplasm. We used a large sample size and a variety of tumour clinicopathological features in lung cancer to investigate the Kaiso expression profile and the relationship between it and those factors. There was a correlation between Kaiso expression and clinicopathological variables in 294 individuals with advanced non-small cell lung cancer (NSCLC). Kaiso expression was examined in 50 lymph node metastases for comparisons between original lung cancer and similar instances of lymph node metastasis. On paraffin-embedded partial lung cancer samples five years ago, immunohistochemistry was utilised for evaluation of Kaiso expression and faster prognostic information. Kaiso's effect on lung cancer patient prognosis was studied using follow-up data. Kaiso was eliminated from three lung cancer cell lines by researchers to understand more about how it contributes to the formation of lung cancer and how it regulates cell proliferation and invasiveness. They next looked at changes in matrilysin transcription and cell proliferation and invasiveness, all of which were found to be normal. The paper is organized as follows.  Section 1 depicts the basic introduction to Kaiso protein and lung cancer. The related existing methodologies are depicted in Section 2. The problem definition is depicted in Section 3. The technique used for analysing the Kaiso protein production in association with lung cancer is defined in Section 4, while the findings and explanations for the study are in Section 5. Section 6 ultimately summarises the paper.

## 2. Related Works

[1] In p120ctn-deficient lung cancer cells, restoring p120ctn-1A or p120ctn-3A levels restored both cyclin D1 and cyclin E levels. Cyclin D1 cotransfection with p120ctn isoforms 1 or 3 restored cyclin E expression but not p120ctn isoforms 1 or 3, suggesting that p120ctn isoforms 1 or 3 must upregulate cyclin E. When p120ctn-1A was overexpressed, it increased the expression of *β*-catenin and cyclin D1 in an unanticipated way [2]. The *β*-catenin promoter region contains Kaiso binding site sequences and CpG islands that may interact with Kaiso, as determined by bisulfite sequencing PCR on SPC-A-1 and LTEP-a-2 lung cancer cell lines. *β*-Catenin mRNA expression was substantially increased in lung cancer cell lines by 5-Aza-2′-deoxycytidine, a demethylating reagent, but was significantly decreased by Kaiso transfection. This, however, had no effect on the following increase in *β*-catenin messenger RNA production caused by 5-Aza-2′-deoxycytidine. Reference [3] demonstrated by immunoprecipitation that lung cancer cell p120-catenin isoform 3 interacts with the Kaiso protein. Kaiso exited the nucleus through p120-catenin isoform 3 as verified by nuclear-cytoplasmic extraction and immunofluorescence. By inhibiting chromosomal area maintenance-dependent nuclear export with leptomycin, the cytoplasmic enrichment of Kaiso caused by p120-catenin isoform 3 was completely reversed. Tumour tissues and pulmonary cell lines showed greater phosphorylated serine 288 concentrations. Serine 288 phosphorylation in p120-catenin isoform 3 further increased Kaiso binding [4]. In this case, the CCK-8 test was used to determine whether or not there was any suppression of proliferation. The Transwell test was performed to look for indications of migration and invasion. Immunofluorescence labelling was used to investigate the expression and distribution of p120ctn and Kaiso. The researchers used a Western blot test to check for changes in the expressions of p120ctn, isoform 1A, phosphorylation of S288, and Kaiso [5]. According to studies, Kaiso binds to the cyclin D1 promoter and is controlled by the consensus Kaiso binding site (KBS) and methylation CpG-dinucleotides (mCpG). A core KBS near the methyl-CpG sites seems to stabilize Kaiso DNA binding to the cyclin D1 promoter in addition to the methyl-CpG sites [6]. At both sites, chromatin immunoprecipitation (CIP) and electrophoretic mobility shift assays (EMSA) were used to demonstrate Kaiso's binding in vitro (ChIP). Immunofluorescence microscopy was used to look for a Kaiso-specific antibody in adult mice's skin, small intestine, mammary glands, and urine bladder. Kaiso has been found in virtually every tissue that has been studied for the first time. Kaiso has been discovered in the nuclei of virtually every tissue. However, it was mostly cytoplasmic in the photoreceptor cells of the eye (rods and cones). Kaiso is expressed by a small percentage of male germ cells that have been labelled with PLZF and Bmi-1. The initial steps in the epithelial to mesenchymal transition include epithelial cadherin depletion and aberrant connections between p120-catenin (p120ctn), its adherens junction binding partner, and the MUC1-CT cytoplasmic tail, according to researchers [7]. When smoke was present, the MUC1-CT inhibitory peptides PMIP and GO-201 blocked the entrance of p120ctn and MUC1-CT into the nucleus [8]. The function of Kaiso in the development of different malignancies was examined, as well as the potential that Kaiso is a factor in racial inequalities in cancer incidence and/or prognosis [9]. With the help of quantitative automated image processing and computational techniques, we investigated the subcellular distribution of the multifunctional transcriptional regulator Kaiso (ZBTB33) in the tumours of a large racially heterogeneous breast cancer cohort from a designated health disparities area in the United States. In a multivariate analysis of the relationship between Kaiso's subcellular distribution and other breast cancer indicators, the researchers discovered new functional and predictive links between Kaiso and the autophagy-related proteins LC3A/B, which have previously been linked to tumour immune microenvironment characteristics, survival, and race in previous research. According to these findings, Znf131 may have a function in the activity of the Kaiso-mediated enzymes [10]. Znf131 may be found in the promoters of many of Kaiso's target genes, including CCND1, which is a transcription factor. A ChIP study of HCT116 and MCF7 cells revealed that the Znf131/Kaiso heterodimer bound to a region that included the previously known +69 Kaiso binding site, which was previously published. Human umbilical vein and microvascular endothelial cells from newborns have been found to be resistant to cell death when treated with the compound Kaiso (HMEC-1s). When Kaiso was overexpressed, hydrogen peroxide was able to reduce apoptosis while simultaneously increasing cell viability. Kaiso was able to raise B-cell CLL/lymphoma 2 (BCL2) levels while simultaneously reducing BAX and BCL2-interacting killer levels by changing gene promoter activity (BIK). Methylation DNA and a specific Kaiso binding site contributed to Kaiso's ability to regulate gene expression in a positive manner (KBS). In the cytoplasm, AKT1 phosphorylates T606-threonine (which is found in the Kaiso RSSTIP motif), according to this research [11–13]. When the pT606-Kaiso and T606A mutations were removed from Kaiso, a significant portion of its 14-3-3 protein-connecting capacity was lost. It was also necessary for pT606-Kaiso to accumulate in the cytoplasm for the link between Kaiso and P120ctn to be established. It has been shown that overexpression of Kaiso target genes such as CDH1 by pT606-Kaiso increases cytoplasmic accumulation and inhibits transcription of Kaiso target genes, respectively. The meaning of the E535 and CH3 codes is examined in more detail. We were able to determine the structures of the wild-type Kaiso (WT) and E535 mutants, as well as their interactions with methylated DNA, via the use of NMR, X-ray crystallography, and in vitro protein-DNA binding studies. It has been discovered that the transcriptional repressor KIS (transcriptional silencing factor) interacts with the methylated miR-200 family and that treatment with 5-Aza prevents this interaction from occurring. Sh-Kaiso PC-3 cells have increased expression of microRNAs (miR) 200a/b/c, miR-141, and miR-429, with miR-200c exhibiting the highest increase [14, 15]. This study made use of two distinct transgenic mouse lines that express varying quantities of the Kaiso gene in order to better understand the underlying processes of the Kaiso-mediated intestinal inflammatory phenotype, which was discovered by the researchers (KaisoTg). KaisoTg mice's intestines were found to have IBD-like damage, including mucosal thickening, gut “lesions,” and crypt abscesses, according to the research. miR-4262's biological effects and activities in CC cell lines were studied in [16] to get a deeper understanding. Cells and tissues from cancer patients had lower miR-4262 levels compared to non-CC patients'. Kaiso (ZBTB33), a BTB/POZ family member, was shown to have a negative correlation with miR-4262 expression in both CC tissues and cell lines. By causing intestinal inflammation and increasing populations of all three secretory cell types when the Kaiso gene was overexpressed, the researchers concluded that Kaiso is a modulator of Notch signalling in the guts. It is critical to determine Kaiso's function in Notch signalling and the link between the KaisoTg secretory cell fate phenotype and Kaiso-induced inflammation [17, 18]. TNBC tissue blocks from Nigeria and Barbados were used to construct a Nigerian/Barbadian tissue microarray, which was then analysed (NB-TMA). In this NB-TMA as well as in a commercially accessible TMA that included both AA and CA TNBC tissues, immunohistochemistry was used to determine if Kaiso expression was associated with clinical features of TNBC (AA-CA-YTMA) [19, 20]. The expression of miR-143-3p in lung cancer is low, while the expression of CTNND1 is high. miR-143-3p inhibited cell growth and invasion by inducing cell death and increased the production of the Bax protein, which is a transcription factor. CTNND1 was found to be suppressed, which caused the cellular properties to change in the other way. According to the results of the dual luciferase reporter experiment, miR-143-3p is a CTNND1 target. Patients with triple-negative breast cancer were studied to determine the impact of Kaiso depletion on cell proliferation and survival [21]. It is unclear what causes fibrillary glomerulonephritis (FGN), a disease with a dismal prognosis. It has been necessary to demonstrate glomerular deposition of Congo red negative and IgS staining antisera-positive fibrils by electron microscopy till recently to diagnose this illness. FGN's new proteomic tissue biomarker, DNAJB9, was identified by us lately [22]. Kaiso is a transcription regulator that binds to snippet Kaiso binding or methyl-CpG dinucleotides. It belongs to the BTB or POZ transcription factors protein family [23]. More than 600,000 new lung cancer diagnoses were expected to be made each year in China in 2010, according to statistics from the National Central Cancer Registry (NCCR), which reported a 46.08 per 100,000 population crude incidence of lung cancer in 2010 (416,333 males and 189,613 females). In contrast, China's lung cancer mortality rate was 37.00 deaths per 100,000 people (50.04 per 100,000 men and 23.33 per 100,000 women). Adenocarcinoma is the most common lung cancer subtype in China, which is consistent with the trend in industrialised nations. Patients with adenocarcinoma, which is the most common form of advanced non-small cell lung cancer (NSCLC), are increasingly receiving targeted therapy. Accordingly, in that study, we outline the epidemiology of NSCLC driver genes based on the extensive work done by Chinese researchers in this area. We hope this will assist clinicians better screen particular driver genes for treatment options in China.

## 3. Proposed Work

### 3.1. Tissue Samples

**Figure 1:**
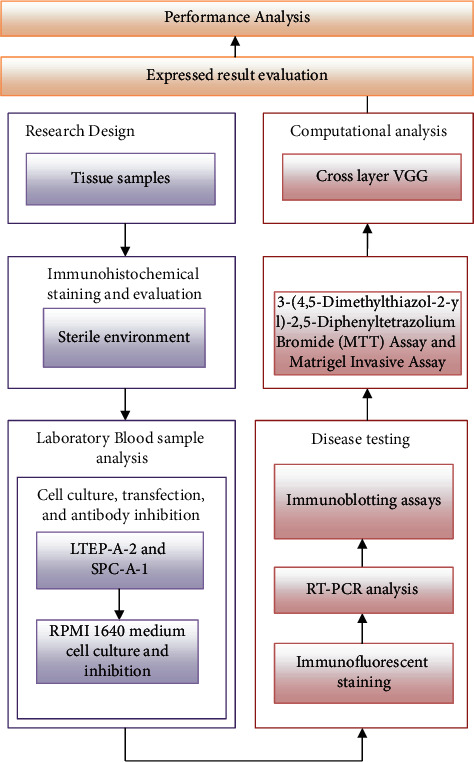
Schematic representation of the suggested methodology.

Between 2018 and 2021, China Medical University's First Affiliated Hospital operated on 294 patients with NSCLC and collected tumour samples thereafter. For protein analysis, 20 of the 294 samples were rapidly frozen in liquid nitrogen and kept at −70°C. Tumours and nontumour components were among the samples (at least 5 cm distant from the primary tumour boundary). Due to publicly accessible data, only 50 of the 294 individuals who had lymph node metastases could be identified. Prior to tumour excision, none of the patients had had radiation, chemotherapy, or immunotherapy. The patients were divided into 165 males and 129 women, resulting in a male to female ratio of 1.87 : 1. On average, patients ranging in age from 35 to 81 years had surgery. The tumours were classified using the TNM stage of the International Union Against Cancer (UICC updated) in 2020. All specimens were reevaluated for diagnosis in line with WHO classification criteria, and 133 instances of lung cancer diagnosis were confirmed, with 146 cases of lung cancer diagnosis confirmed as adenocarcinomas. For immunohistochemical analysis of 50 samples with autologous lymph node metastases, twenty-one autologous lymph node metastases, twenty-three autologous lymph node metastases, and six autologous lymph node metastases were used as matched samples (Figure 1).

### 3.2. Immunohistochemical Staining and Evaluation

Slices having a thickness of 4 millimetres are obtained by slicing through formalin-fixed, paraffin-embedded specimens. The dewaxed sections were autoclaved for 105 seconds in a citrate buffer after being rehydrated in ethanol step by step (pH 6.0). Nonimmune sera and H_2_O_2_ at a concentration of 3% decreased endogenous peroxidase activity, whereas nonspecific binding was prevented. Sections were incubated with primary antibodies for another day at room temperature before being sent to the lab. Santa Cruz Biotechnology was used by the researchers. Upstate 4 g/ml mouse anti-human Kaiso monoclonal anti-bodies and 4 g/ml mouse anti-human Kaiso polyclonal anti-bodies (C18). After that, secondary anti-biotinylated antibodies were stained for another day (Maixin Biotechnology, Fuzhou, Fujian, China). The 3,3′-diaminobenzidine tetrahydrochloride was used to initiate the peroxidase reaction (Maixin Biotechnology). Before mounting, the sections were washed in alcohol and counterstained with hematoxylin. After that, the pieces were dried thoroughly before being mounted. For the negative control, primary antibodies were utilised instead of PBS. All of the stained sections were assessed by observers who were unaware of the patients' clinical condition: SDDD, YW, and EHW. When there were disputes, the investigators reevaluated the cases and came to an agreement. The sections were examined at a low magnification (100x) for areas where Kaiso staining was consistent. After counting 400 tumour cells, we looked at the percentage of cells that stained positively. Kaiso expression was detected in the following percentages of cells: 0 indicates less than 25%, 1 represents 26–50%, 2 represents 51–75%, and 3 represents more than 75%. Based on the relative intensity of the staining, it was rated from 1 (weak) to 3 (moderate) (strong). Researchers combined the percentage and intensity data to arrive at a final score. In order to obtain final statistical results, any score less than one was considered negative, while any score of two or more was deemed positive. When at least 5% of the cells in the cytoplasm and nucleus tested positive for anti-Kaiso antibody, it was concluded that the case was nuclear-positive.

### 3.3. Cell Culture, Transfection, and Antibody Inhibition

BE1 cells were isolated from a patient with lung giant cell cancer. The Chinese Academy of Sciences' Cell Bank supplied the LTEP-A-2 and SPC-A-1 human lung cancer cell lines (Shanghai, China). For 48 hours before collection, cells were cultured in RPMI 1640 medium (GIBCO Inc., Los Angeles, CA, USA) supplemented with antibiotics (I.U./ml penicillin and streptomycin) (Sigma). We received three Kaiso shRNA plasmids from Open Biosystems (RHS1764-9214280, RHS1764-9216302, and RHS1764-9692262), as well as a control plasmid (pSM2 shRNAmir) that does not mutate any genes (RHS1707). The pSHAG-MAGIC2 vector was inserted using the following sequences:

1. TGCTGTTGACAGTGAGCG AGGCAGTTATTAGGAGTGAAATTAGTGAAGCCACAGATGTAATTTCACTCCTAATAACTGCCC TGCTGTTGACAGTGAGCG AGGCAGTTATTAGGAGTGAAATTAGTGAAGCCACAGATGTAATTTCACTCCTAATAACTGCCC TGCTGTTGACAGTGA
2. TGCTGTTGACAGTGAGCG TGCCTACTGCCTCGGA TGCCTACTGCCTCGGATGCCTACTGCCTCGGATGCCTACTGCCTCGGA TGCCTACT AGGTCAGAAGATCATTACTTTATAGTGAAGCCACAG 3. ATGTATAAAGTAATGATCTTCTGACCC TGCCTACTGCCTCGGA
3. TGCTGTTGACAGTGAGCG TGCTGTTGACAGTGAGC CGCCGTTACTGTGAGAAGGTATTAGTGAAGCCACAGATGTAATACCTTCTCACAGTAACGGCA TGCCTACTGCCTCGGA TGCCTACTGCCTCG (bold Codes indicate the shRNA plasmids' sense, loop, and antisense sequences).

In accordance with the manufacturer's instructions, transfections were carried out using the Arrest-In^TM^ Transfection Reagent from Open Biosystems (USA). The transfected cells were harvested and analysed after a transfection window of 48 hours. Because of its relative efficacy and durability, we chose the second shRNA plasmid during our pilot testing over the first. One particular monoclonal antibody, mAb 6F from the University of Rochester Medical Center (Upstate), was added to the growth medium and maintained at a final concentration of 100 ng/ml throughout the study as a part of the validation procedure. Beijing Zhongshan Golden Bridge Biotechnology Co. provided mouse anti-human IgG at a final concentration of 100 ng/ml to the corresponding control groups (Beijing, China).

### 3.4. Immunofluorescent Staining

Immunofluorescent staining was meticulously followed to guarantee precision. To make the cells permeable, they were first fixed for 15 minutes in cold, 100% methanol (−20°C) before being permeabilized with 0.2% Triton-X100 (pH 8.0). C-18 and two mouse monoclonal antibodies (4 g/ml each; Upstate in Lake Placid, New York, and Santa Cruz Biotechnology Inc.) were used to detect Kaiso with polyclonal antibodies and cells overnight at 4°C in the lab. Incubation of an anti-rabbit secondary antibody coupled to the fluorescent dye rhodamine/fluorescein isothiocyanate followed the incubation of the main antibody (FITC) (Beijing Zhongshan Golden Bridge Biotechnology Co.). Color comparison of nuclei was accomplished using PI (50 mg/mL; Sigma) to highlight differences in nuclei's colour. The cells were studied in Tokyo, Japan, using an Olympus IX51 fluorescent microscope and a Coolpix 5400 camera (Nikon, Japan).

### 3.5. RT-PCR Analysis

With the use of the TRIzol reagent, we were able to extract whole RNA (Invitrogen). To synthesise cDNA, we followed the manufacturer's instructions and utilised the RNA PCR Kit (AMV) Version 3.0. TAKARA BIO INC. is headquartered in Dalian, Liaoning, China. Additionally, we considered the linear amplification range, annealing temperatures, and number of PCR cycles in addition to the primer sequences. A 15 percent agarose gel with 0.1 micrograms of ethidium bromide per millilitre was run on the Bioimaging System for PCR analysis. The device for Bioimaging was from UVP, Upland, CA, USA. Each band's grayscale intensity value had to be normalised to *β*-actin before the transcriptional level of each gene could be determined. In order to get the most precise findings, each experiment was repeated five times.

### 3.6. Immunoblotting Assay

To remove the lysate buffer, the cells were homogenised on ice with a lysate solution comprising 20 mM Tris-HCl, 1 mM EDTA, 50 mM NaC, 50M NaF, 1 mM Na3VO4, and 1 percent Triton-X100 (PBS). For 30 minutes at 15000 rpm, the homogenate was centrifuged at 4°C at a speed of 4000*g*. When the recovered supernatant was tested for protein content, the results were recorded using a BCA method (BCA protein assay kit23227, Pierce Biotechnology). Each sample produced 80 grams of total protein using an 8 percent SDS-PAGE and PVDF membranes for protein blotting. After SDS-PAGE analysis, the total protein extracts were immunoblotted using the aforementioned antibodies. Antibodies against Kaiso and *β*-actin polyclonal were utilised in immunoblot experiments (a housekeeping protein used as a loading control to assure equal amounts of protein in all lanes). 0.05 percent Tween-20 was added to TBS (pH 7.5) to prevent nonspecific binding. Antibodies against Kaiso (1 : 1000, C-18; Santa Cruz Biotechnology Inc.) and *β*-actin (1 : 200; Beijing Zhongshan Golden Bridge Biotechnology Co., Beijing, China) were incubated overnight at 4°C (TBST). After a 2-hour incubation at 37°C with secondary antibodies (1 : 2000, ZDR-5306) tagged with horseradish peroxidase after third-generation TBST washing (all from Zhongshan Biotechnology), these straps were identified by using the DAB Kit-0031 by Maixin Biotechnology. ImageJ was used to analyse the images captured by the Bioimaging System bands (Upland, CA, USA). The *β*-actin and interest protein optical densities were computed for each sample and shown graphically as a ratio.

### 3.7. 3-(4,5-Dimethylthiazol-2-yl)-2,5-Diphenyltetrazolium Bromide (MTT) Assay and Matrigel Invasive Assay

ShRNA-Kaiso, Kaiso antibody addition, and control cells were all seeded in the same 96-well plate, with 5000 of each type in each well. For four days following the MTT treatment, researchers monitored cellular development in the lab's animal cells. A microplate reader was used to count the number of live cells in each well since the absorbance at 570 nm is directly proportional to the number of cells in that well (Model 550, Bio-Rad, Hercules, CA, USA). All studies utilised an active component control sample, and the results were compared to those of that sample (DMSO). New York-based Corning Inc. tested the invasiveness of the cells according to the manufacturer's procedure using Transwell inserts with an 8-meter polycarbonate membrane. Thin layers of Matrigel (1 : 4 dilution) were injected on the membrane's top side. Cell suspension (5, 105/ml) was added to the Matrigel-lined top compartment after it had hardened. This experiment's chemoattractant was 10% FBS-supplemented media. After 48 hours of cultivation, noninvading cells were removed from the filter surface using a cotton brush. After that, the filters were stained with hematoxylin. Five randomly chosen 200-cell regions were counted under an inverted microscope using cells that came from the filter's underside (the Olympus IX51, manufactured by Olympus America Inc. in Melville, New York). There were three exams in all, and two of them were done a third time to ensure accuracy.

### 3.8. Computational Analysis

Finally, the classification is used to choose the biomedical data sets for inference after the biological analysis. The cytoplasmic expression of Kaiso was related to clinical and pathological factors. It was possible to compare the cytoplasmic Kaiso expression in original tumours and lymph node metastases. For the most part, this step involves classifying terms using the cross-layer VGG. A difference between two independent variables may be easily calculated in this way. With the use of odds and a function, CLVGG may be computed. In other words, the dispensation is collected in this manner. In order to classify the data, CLVGG must first analyse and redistribute it. Only then will it utilise its class probability to do so. With equation (1), it is possible to start the cycle by activating the neuron.

(1) $$\begin{array}{c} K_{n_{j}}^{l}=\sigma \sum_{K}x_{jk}^{l}a_{K}^{l}+b_{j}^{l}. \end{array}$$

Equation (2) illustrates vectorized forms for expressing the equation:

(2) $$\begin{array}{c} b_{j}^{l}=\sigma \left(x^{l}a^{l-1}+b^{l}\right). \end{array}$$

Equation (3) depicts the quadratic set into which the training set may be merged:

(3) q=12Y−al2=12∑jyj−ajl^2.

Equations (4) and (5) provide the gradient survival output.

(4) $$\begin{array}{c} \frac{\partial ∁}{\partial w_{kj}^{l}}=ak^{l-1}\delta_{j=C}^{l}, \end{array}$$

(5) $$\begin{array}{c} F=\det\left[sn\right]-k{\left(\text{classify}\left(sn\right)\right)}^{2}, \end{array}$$

where *F* is the feature, *s* is the survival rate, and *n* is the probability. These are to be declared as follows:

(6) $$\begin{array}{c} \det\left[sn\right]=a_{j}^{l}b_{j}^{l}, \end{array}$$

(7) $$\begin{array}{c} \text{Probability}\left(sn\right)=a_{j}^{l}b_{j}^{l}. \end{array}$$

The classification is concluded in the following equation:

(8) $$\begin{array}{c} F=a_{j}^{l}b_{j}^{l}-m{\left(a_{j}^{l}b_{j}^{l}\right)}^{2}, \end{array}$$

where *m* is the survival constant. The overall process of the comparison of the cytoplasmic expression of Kaiso and its stage was illustrated by using the suggested cross layer VGG. The steps of cross layer VGG is illustrated in the Pseudocode 1.

   Input: key test data
  Output: Computational analysis
   Step 1: read the input
  Step 2: initialize the data length
  Step 3: Class = dec2bin(raw_data, *C*_*D*_)
  Step 4: Classified _data for *i* = 1:size(,1) for *j* = 1:size _ch = randi(2,size())2 for this value (,2)
     end
     end
  5 Survival rate € = mod(ch + msg,2)/
     € = €()'
  round((1 + randi)^*∗*^N), length, €(:), :, :, :)
  D B is indicating that = randi(2,1)2 is true.
  Sorting newly acquired information (data)
    € =  should be reduced.
  as long as I is more than or equal to one. file probability method = (1, file drop)
  the message (i,j) bin _msg = str2num
     end
     end
     End

## 4. Performance Analysis

Both healthy lung epithelium and invasive lung carcinoma expressed the Kaiso gene. Adult bronchial epithelial cells and glands in normal tissue had the amino acid Kaiso in their cytoplasm. Kaiso expression was shown to be more prevalent in lung cancer metastases than in the primary tumour.

First, we looked for Kaiso expression in the cells since it has been recently linked to a poor prognosis in non-small cell lung cancer. As long as Kaiso was expressed differently in different lung cancer histological subtypes, no other clinicopathological features were found to be associated with it.

**Figure 2:**
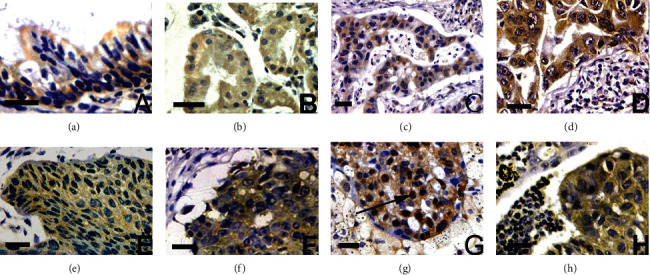
Disease expression.

The apiculus of these cells and a few glands were the only places where Kaiso expression was detected in all 20 normal lung tissues (Figures 2(a) and 2(b)). As a result of our evaluation criteria, they were classified as negative expressions of emotion. Cancer cells that had been labelled favourably exhibited high levels of Kaiso, which was found mostly in their cytoplasm (Figures 2(c)–2(f)). It serves as a platform for other organelles in the cell to function. The cytoplasm of a cell performs all tasks related to cell proliferation, growth, and reproduction. A cell's semifluid cytoplasm is also referred to as the protoplasm's nonnuclear content, since it is located both outside and within the nuclear envelope. Tumour cells were sometimes stained with nuclear antigen, although this happened very rarely (15/294 times; 50 percent of the time) (Figure 2(g)). No correlation was found between this staining pattern and any medical conditions. An additional monoclonal antibody confirmed the Kaiso subcellular labeling's precision, C-18. This is much higher than the normal bronchial epithelium (*p* < 0.001), with 63.61 percent of Kaiso's cytoplasmic expression positive (187/294).

**Figure 3:**
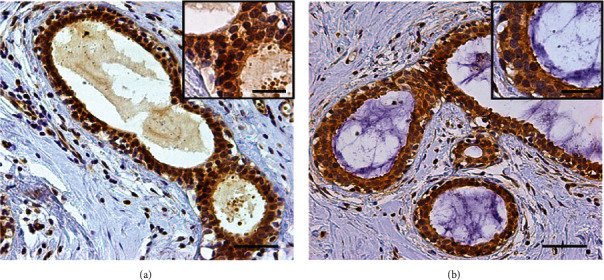
Normal and abnormal level of Kaiso expression.

**Figure 4:**
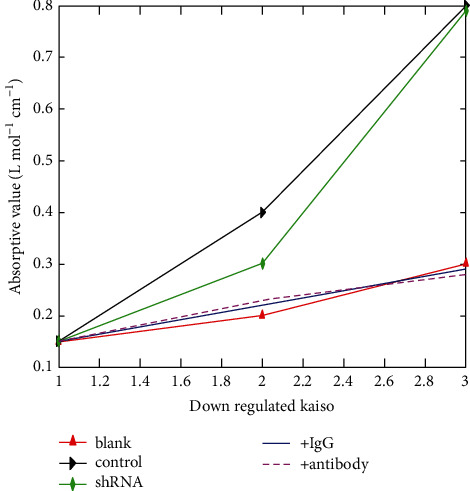
Downregulated Kaiso versus absorptive value.

Normal breast epithelium shows variable Kaiso expression (Figure 3), with higher levels in the nuclei Figure 3(a) than in the cytoplasm Figure 3(b) (Figure 4).

**Figure 5:**
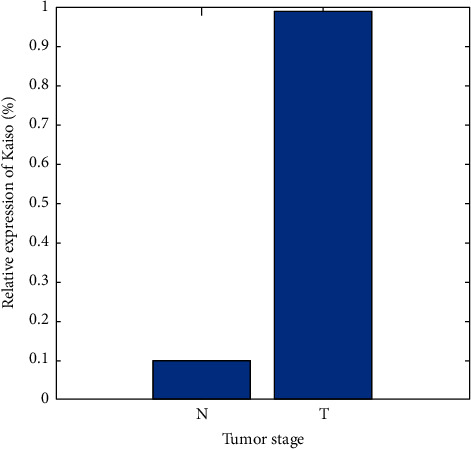
Tumour stage versus relative expression of Kaiso.

As shown in Figure 4, clear Kaiso downregulation promotes matrilysin transcription and lung cancer cells' capacity to proliferate and invade. BE1, LETP-A-2, and SPC-A-1 cells transfected with shRNA-Kaiso or treated with a Kaiso antibody underwent invasion tests. The number of cells infiltrating the bottom surface of the filter 48 hours after plating on Matrigel was much higher in the control group. The results obtained should be varied absorptive value for the different proteins. SD is shown as error bars next to the columns that show the mean (*n* = 3) (Figure 5).

**Figure 6:**
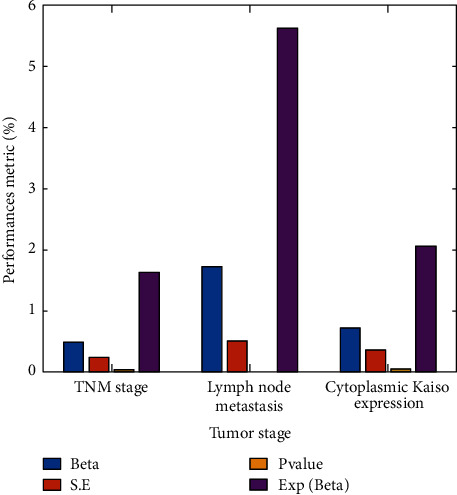
Tumour stage versus performance metrics.

Kaiso protein expression in matched tumourous (T) and nontumourous (N) tissues from eight of twenty NSCLC patients is shown in the examples below. There are two lanes: one for tumour tissue and one for normal lung tissue that surrounds the tumour. As can be seen from the band intensities, there is a substantial increase in Kaiso expression in tumourous tissue as compared to normal tissue from the same patient. To ensure that all lines had the same quantity of protein, *β*-actin was utilised as a loading control. When Kaiso was compared to *β*-actin, the optical density ratio was determined and shown visually. Tumourous (T) versus nontumourous (N) tissues were compared statistically for Kaiso expression. Neoplastic tissues had higher Kaiso immunoreactivity (*p* ≤ 0.001). The results were summarised as follows: mean plus or minus SD. The columns represent the average (based on a sample size of 20 people), while the error bars represent the standard deviation (Figure 6).

**Figure 7:**
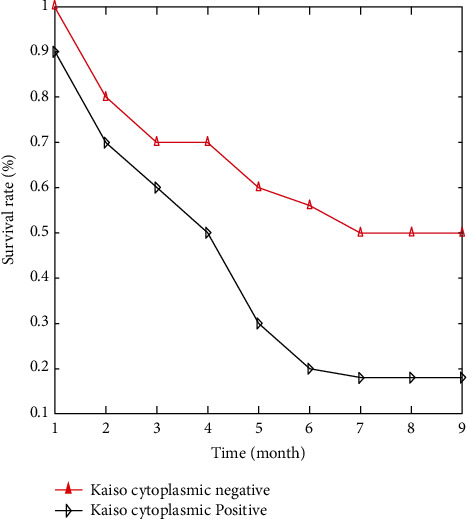
Time versus survival rate.

A multivariate Cox regression analysis was performed to examine the cytoplasmic expression of Kaiso as a prognostic factor further. Lymph node metastases and tumour stage were both significant (*p*=0.001); however, neither was associated with patient outcome (*p*=0.047), as can be shown in Figure 6. It is also possible that the cytoplasmic Kaiso status is an independent predictor of prognosis (Figure 7).

The overall five-year survival rate for lung cancer patients was 34.1%, with cytoplasmic Kaiso positivity occurring in 22.2% of patients and cytoplasmic Kaiso negativity occurring in 64.00% of patients. Kaiso cytoplasmic expression was shown to be associated with a low overall survival rate in a single-factor analysis. In patients with cancers that expressed the Kaiso gene, the survival rate was substantially lower than that in individuals with tumours that did not express the Kaiso gene (*p*=0.002; Figure 5).

## 5. Conclusions

Kaiso was mostly expressed in the cytoplasm of lung cancer tissues, and this was associated with an advanced TNM stage, lymph node metastases, and a poor prognosis for patients. However, further research is needed to determine if Kaiso can be utilised as an independent prognostic predictor (*p*=0.054, Cox multivariate analysis). For a number of lung cancer cell lines that had been grown in vitro, Kaiso was mostly found in the nucleus. To enhance the expression of the suppressed gene matrilysin and stimulate cell proliferation and invasion, we used shRNA-Kaiso and Kaiso-specific antibodies in combination. There are still many questions to be answered, but early results show that the various locations of Kaiso in the cytoplasm and the nucleus have distinct biological roles. We also found cells expressing Kaiso in both the cytoplasm and nucleus, despite the fact that it is mostly found in the nucleus in cultivated cells. To better understand the biological functions of cytoplasmic and nuclear Kaiso, plasmids enhancing cytoplasmic and nuclear Kaiso localization will be useful in the future. As a result, research like these will help identify the precise pathways that link cytoplasmic Kaiso expression to lymph node metastases as well as a bad prognosis.

## Data Availability

The data used to support the findings of this study are available from the corresponding author upon request.
